# Transition From Epstein-Barr Virus (EBV)-Positive Rectal Hodgkin Lymphoma to Diffuse Large B-Cell Lymphoma in the Lung

**DOI:** 10.7759/cureus.65013

**Published:** 2024-07-20

**Authors:** Jing Di, Sainan Wei, Amie Jackson, Reinhold Munker, Melissa V Kesler

**Affiliations:** 1 Pathology and Laboratory Medicine, University of Kentucky College of Medicine, Lexington, USA; 2 Hematology and Oncology, Baptist Health Lexington, Lexington, USA; 3 Hematology and Oncology, University of Kentucky HealthCare, Lexington, USA

**Keywords:** hodgkin lymphoma, lung, rectum, diffuse large b-cell lymphoma, ebv

## Abstract

We report a distinctive case of sequential lymphomas in a 72-year-old male, initially diagnosed with Epstein-Barr virus (EBV)-positive rectal classic Hodgkin lymphoma (cHL), followed by the development of diffuse large B cell lymphoma (DLBCL) in the lung. This rare progression underscores the complexity of lymphomas associated with EBV infection and their unpredictable clinical courses. The patient's journey began with symptoms of intractable diarrhea, low appetite, and significant weight loss, leading to the diagnosis of stage 4B cHL, managed initially with brentuximab/doxorubicin, vinblastine, dacarbazine (AVD) chemotherapy. Despite a partial response, surveillance identified a transition to DLBCL, marked by new pulmonary lesions. This case highlights the clinical and diagnostic challenges in managing sequential lymphomas, emphasizing the role of EBV in lymphomagenesis and the potential for clonal evolution from a common precursor cell. The therapeutic approach evolved from targeted chemotherapy to consideration of advanced treatments such as autologous stem cell transplant and chimeric antigen receptor (CAR) T-cell therapy, reflecting the aggressive nature and poor prognosis of the disease. This case contributes to our understanding of the EBV's impact on lymphoma progression and underscores the need for vigilant monitoring and adaptive treatment strategies in similar clinical scenarios.

## Introduction

The interaction between viral infections and lymphomagenesis presents a complex area of study within oncology, exemplified by the association between Epstein-Barr virus (EBV) and various lymphomas [1-3]. EBV, a ubiquitous herpesvirus, is linked to a range of lymphoproliferative disorders, including both Hodgkin lymphoma (HL) [4] and non-HL (NHL), such as Burkitt lymphoma and EBV-positive diffuse large B-cell lymphoma (DLBCL) [1]. The case presented herein illustrates the rare and intriguing occurrence of sequential lymphomas, initially presenting as EBV-positive rectal HL, followed by the emergence of DLBCL in the lung.

EBV’s role in oncogenesis involves genetic, immunologic, and environmental factors contributing to malignant transformation [1]. The progression from HL to DLBCL or vice versa, particularly in the context of EBV infection, raises intriguing questions about the clonal relationship between these lymphomas and the role of EBV in their pathogenesis [1,5]. The "hit-and-run" hypothesis suggests that EBV may initiate oncogenic processes and become dispensable for the maintenance of the malignant phenotype, potentially explaining the emergence of sequential lymphomas with different EBV statuses [6,7]. Additionally, the phenomenon of clonal evolution, where a common precursor cell gives rise to different lymphoma subtypes through divergent genetic and epigenetic changes, may play a role [8,9].

The incidence of sequential lymphomas, where a patient develops one type of lymphoma followed by another distinct subtype, is rare, with an occurrence rate varying between 1% and 4.7% across different studies [5,10], and the occurrence within the same individual of HL followed by DLBCL is exceptionally uncommon. This case highlights the clinical challenges and therapeutic considerations in managing such patients. The intricacies of diagnosing, treating, and understanding lymphomas associated with EBV infection necessitate a multidisciplinary approach, incorporating advances in molecular genetics, immunology, and targeted therapies.

## Case presentation

**Figure 1:**
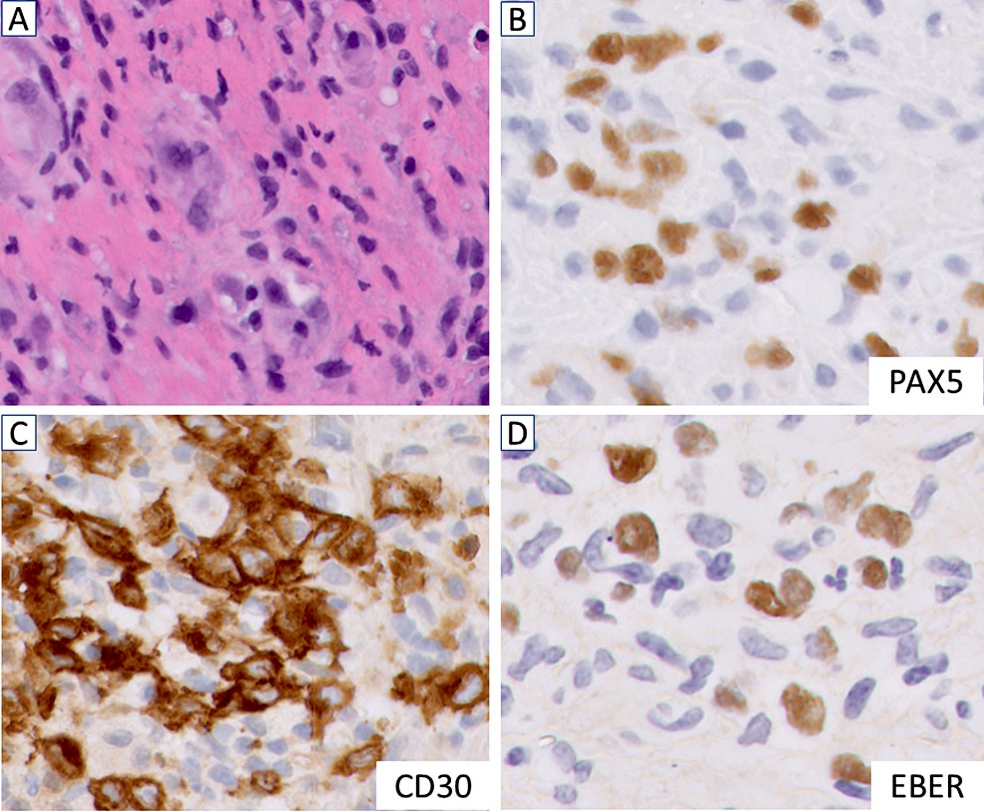
Histopathological examination of the rectal tissue showed features of classic Hodgkin lymphoma. A. Hematoxylin and eosin (H&E) stain showed the characteristic cellular composition. B. Immunohistochemistry for PAX5 with dim positive staining in some cells. C. CD30-positive cells indicate atypical large cells typically found in Hodgkin lymphoma. D. Epstein-Barr virus-encoded RNA (EBER) in situ hybridization demonstrating positive cells. Magnification, x40

A 72-year-old male patient presented with persistent diarrhea, diminished appetite, and considerable weight loss, leading to concern for gastrointestinal obstruction. Surgical intervention for a diverting colostomy and tissue biopsies resulted in a diagnosis of stage 4B classic HL (cHL, reviewed and confirmed at the National Institute of Health (NIH)), marked by extensive liver involvement and significant extranodal dissemination. Sections of the rectal biopsies showed a vaguely granulomatous appearance with associated necrosis and a mixed inflammatory infiltrate, including eosinophils and scattered large, atypical cells with prominent nucleoli. Immunohistochemical stains revealed that these large cells were positive for CD30, partial CD15, dim PAX5, and EBER (Figure 1), but negative for CD20, CD45, CD79A, and ALK, confirming the diagnosis.

**Figure 2:**
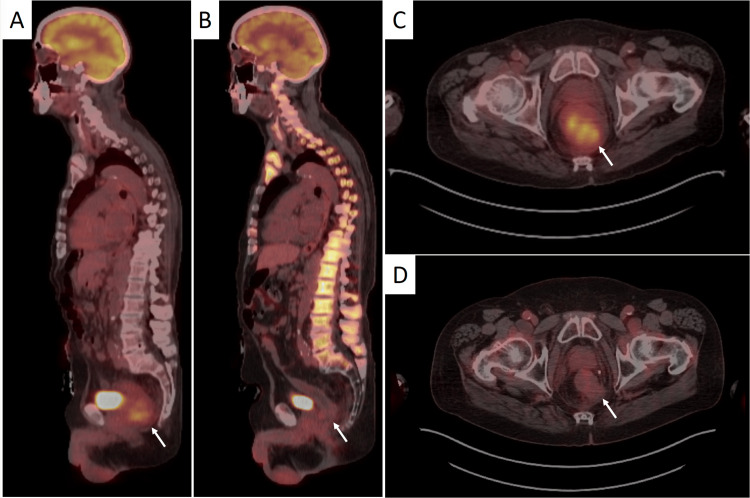
PET scans show the reduction of the rectal mass post-treatment. (A) Sagittal view pre-treatment. (B) Sagittal view post-treatment. (C) Axial view pre-treatment. (D) Axial view post-treatment. White arrows indicate the location of the rectal mass.

**Figure 3:**
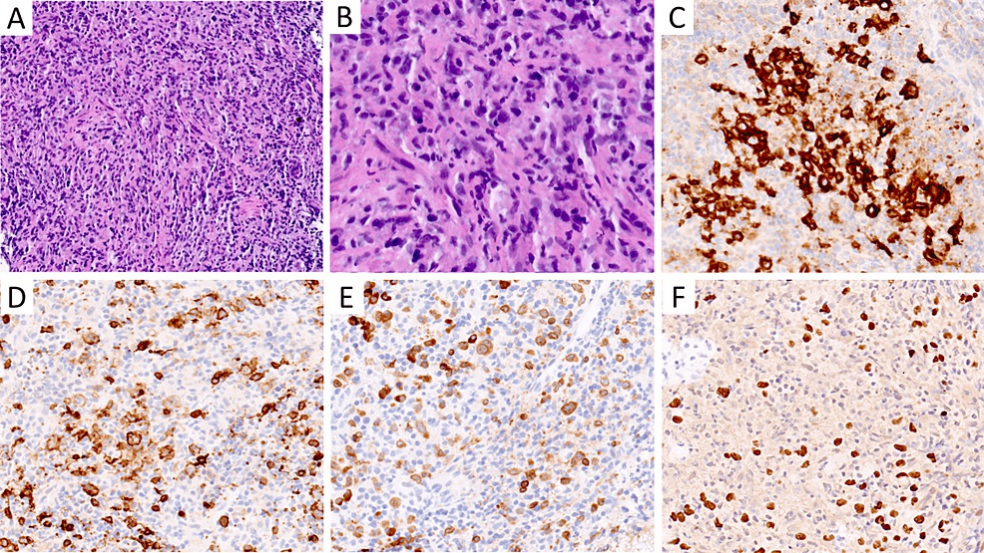
Histopathological examination of the transbronchial biopsy specimen showing features consistent with diffuse large B-cell lymphoma. A&B. Hematoxylin and eosin (H&E) stain revealing atypical lymphoid infiltration; magnifications x20 and x60, respectively. C. Immunohistochemistry highlighting CD30-positive staining in some cells. D&E. CD20- and CD79A-positive staining in atypical large B cells, indicative of B-cell lineage (magnification, x40). F. Epstein-Barr virus-encoded RNA (EBER) in situ hybridization identifying EBER-positive cells (magnification, x40)

The patient underwent six cycles of brentuximab/doxorubicin, vinblastine, dacarbazine (AVD) chemotherapy and radiation, achieving a partial response with a 50% reduction in rectal mass size (Figure 2). However, six months later surveillance, PET scans revealed new pulmonary lesions, indicative of a progression in the lung. Sections of the transbronchial biopsy displayed a different morphology with sheets of large, atypical cells that retained positivity for CD30 and EBER but expressed strong and uniform CD20, CD79A (Figure 3), and PAX5, as well as expressed CD45, BCL6, and MUM1 and negative for CD3 and CD15. Ki-67 highlighted the majority of the large lymphoma cells (at least 85%). This morphologic and immunophenotypic profile was more in keeping with diffuse large B-cell lymphoma, suggesting a possible clonal relationship to the previously diagnosed EBV-positive cHL.

The latest diagnostic evaluations have revealed positive CD19 expression in the patient's lymphoma cells, expanding the potential therapeutic targets. Considering the aggressive nature and refractory course of the disease, the treatment strategy has been escalated to include chimeric antigen receptor (CAR) T-cell therapy. This cutting-edge treatment modality involves genetically modifying the patient's T cells to express CARs targeting CD19-expressing cells. The treatment is currently ongoing, and the patient's response is under close observation.

## Discussion

EBV's role in the pathogenesis of lymphomas is multifaceted and underscores the virus’s oncogenic potential [2,3,6,8,11]. In cHL, the presence of EBV is detected in approximately 40-50% of cases globally, with a higher prevalence in developing countries [8]. EBV is often associated with distinct molecular and immunologic pathways that promote lymphocyte transformation and malignant progression [7]. The clonal relationship between EBV-positive cHL and DLBCL is corroborated by shared EBV positivity, overlapping immunophenotypic markers (CD30 and EBER), and similar histopathological features. This case uniquely illustrates the transition from EBV-positive cHL to DLBCL in the lung, emphasizing the potential for EBV to influence the clonal evolution of lymphocytes.

However, it is essential to consider alternative explanations for this phenomenon [7,10]. This case may represent a transition from one EBV-positive cHL to DLBCL, but it could also involve two unassociated clones that developed at different times [7,10]. Additionally, the development of DLBCL could be secondary to immunosuppression induced by the chemotherapy received for the initial cHL [12]. Additional studies, such as IgH rearrangement or similar mutational analyses, must corroborate or suggest a clonal relationship. Although both lymphomas express CD30 and are associated with EBV, this alone is insufficient evidence to ensure a transition or connection between the two. Acknowledging this limitation is crucial for a comprehensive understanding of the case. Furthermore, the patient’s age, immunosenescence, and the gradual deterioration of the immune system with aging are essential factors. Immunosenescence can increase susceptibility to infections and malignancies, including lymphomas [2]. This decline in immune function could have influenced the development and progression of the initial EBV-positive cHL and subsequent DLBCL, as well as the patient’s overall response to the malignancies and their treatment.

Primary anorectal HL, particularly within the context of HIV-positive patients, represents a profoundly rare clinical entity [8,13-15]. This distinct presentation of HL in the anorectal region is significantly uncommon, with primary colorectal lymphomas constituting only 0.2% of all gastrointestinal lymphomas and primary HL presenting in merely 1-3% of these cases [3,13-17]. A comprehensive review of gastrointestinal lymphoma and its correlation with Hodgkin's disease revealed the incidence of primary rectal HL to be one out of 1,423 cases [13]. Notably, these instances are predominantly observed in HIV-positive individuals or those with associated inflammatory bowel disease (IBD) [13,14,16]. The rarity of this manifestation is underscored through the review of reported cases, including 26 cases of primary rectal-anorectal HL that were adequately reported and published, of which seven cases were exclusively anorectal [13,14].

In the present case, the patient's diagnosis of anorectal cHL in the absence of HIV or other notable immunosuppressive conditions represents a highly atypical and exceptional clinical scenario. The patient was not taking any immunosuppressants and had no known immunosuppressive conditions at the time of diagnosis. Immunodeficiency-associated EBV-positive lymphoproliferative disorder was considered and excluded based on the patient’s medical history and clinical findings. The differential diagnosis between DLBCL and lymphomatoid granulomatosis was clarified by the presence of uniform sheets of large atypical B cells and the absence of angiocentric and angiodestructive patterns, favoring DLBCL [18]. At the time of rectal cHL diagnosis, the patient exhibited systemic involvement, complicating the distinction between primary rectal cHL and systemic disease with rectal involvement. However, the predominant symptoms and initial diagnostic biopsies were from the rectal lesion, leading to the initial identification and treatment focus on rectal cHL.

Notably, the diagnosis of DLBCL with the diffuse expression of EBER could suggest EBV-positive DLBCL according to WHO criteria [18]. However, the criteria for this diagnosis require the exclusion of inborn or acquired immunodeficiency, a history of lymphoma, and other EBV-related lymphomas and lymphoproliferative disorders. This patient presents with all three criteria: acquired immunodeficiency due to immunosuppression induced by chemotherapy for cHL, a history of lymphoma, and a history of EBV lymphoma. Therefore, the diagnosis of EBV-positive DLBCL is excluded. Considering the patient's history of chemotherapy and the presence of EBV, the correct diagnosis, according to WHO, should be DLBCL, EBV+/HHV8-, iatrogenic (after a multi-chemotherapeutic regimen) [18]. This represents a rare and underdiagnosed group of lymphoid malignancies.

The occurrence of sequential lymphomas, particularly transitioning from cHL to DLBCL, suggests a complex interplay of genetic and environmental factors [19]. One hypothesis is that both lymphomas might originate from a common precursor cell that undergoes divergent evolution driven by genetic mutations, epigenetic changes, and viral influences such as EBV [9,19,20]. This case exemplifies the potential for lymphomas to evolve in clinical behavior and histopathology, highlighting the need for vigilant monitoring and adaptability in treatment approaches.

## Conclusions

This case illustrates the intricate interplay between EBV infection and the sequential emergence of lymphomas, highlighting the complexity of these malignancies and the pivotal role of EBV in their evolution. It underscores the need for an in-depth understanding of the biological mechanisms driving lymphoma progression and the critical need for individualized and responsive treatment plans. The recent discovery of CD19 expression in the patient's lymphoma cells and the commencement of CAR T-cell therapy mark a significant turning point in therapeutic management, showcasing modern oncologic care's dynamic and responsive nature.
